# Association study between the monoamine oxidase A gene and attention deficit hyperactivity disorder in Taiwanese samples

**DOI:** 10.1186/1471-244X-7-10

**Published:** 2007-02-28

**Authors:** Xiaohui Xu, Keeley Brookes, Chih-Ken Chen, Yu-Shu Huang, Yu-Yu Wu, Philip Asherson

**Affiliations:** 1 MRC Social, Genetic and Developmental Psychiatry Centre, Institute of Psychiatry, King's College London, London, UK; 2 Department of Psychiatry, Chang Gung Memorial Hospital, Taiwan; 3 Chang Gung University School of Medicine, Taiwan

## Abstract

**Background:**

Attention deficit hyperactivity disorder (ADHD) is a common and highly heritable disorder of childhood characterized by inattention, hyperactivity and impulsivity. Molecular genetic and pharmacological studies suggest the involvement of the dopaminergic, serotonergic and noradrenergic neurotransmitter systems in the pathogenesis of ADHD. Monoamine oxidase A (MAO-A) encodes an enzyme that degrades biogenic amines, including neurotransmitters such as norepinephrine, dopamine and serotonin. In this study we examined a 30 bp promoter variable number tandem repeat (VNTR) and a functional G/T single nucleotide polymorphism (SNP) at position 941 in exon 8 (941G/T) of MAO-A for association with ADHD in a Taiwanese sample of 212 ADHD probands.

**Methods:**

Within-family transmission disequilibrium test (TDT) was used to analyse association of MAO-A polymorphisms with ADHD in a Taiwanese population.

**Results:**

A nominally significant association was found between the G-allele of 941G/T in MAO-A and ADHD (TDT: *P *= 0.034. OR = 1.57). Haplotype analysis identified increased transmission of a haplotype consisting of the 3-repeat allele of the promoter VNTR and the G-allele of the 941G/T SNP (*P *= 0.045) to ADHD cases which the strong association with the G-allele drove.

**Conclusion:**

These findings suggest the importance of the 941G/T MAO-A polymorphism in the development of ADHD in the Taiwanese population. These results replicate previously published findings in a Caucasian sample.

## Background

Attention deficit hyperactivity disorder (ADHD) is one of the most common childhood behavioural disorders characterised by inattention, hyperactivity and impulsivity. Current estimates show that 3%–6% of school age children have a diagnosis of ADHD [1]. Polymorphic variants in several genes involved in regulation of the dopamine, and related neurotransmitter pathways are reported to be associated with ADHD [2,3].

Monoamine oxidase A (MAO-A) is located on the X-chromosome (Xp11.23–11.4) and is comprised of 15 exons. MAO-A degrades biogenic amines such as dopamine and serotonin, providing control of the level of these neurotransmitters in the central nervous system. MAO-A knockout mice have elevated brain levels of serotonin, norepinephrine and dopamine and manifest aggressive behaviour [4]. Caspi et al. [5] reported that maltreated children with a genotype conferring high levels of MAO-A expression were less likely to develop antisocial problems.

Eklund et al. [6] found that low MAO activity and high tri-iodothyronine (T3) level were significantly associated with violent offenders with an early behavioural risk pattern. They further reported that attention difficulties were associated with low MAO activity. Low levels of platelet MAO activity were reported to be associated with attention and impulsivity in boys with ADHD [7].

MAO-A has previously been proposed as a potential susceptibility gene for ADHD and investigated in several association studies, with both positive and negative reports. The short 3-repeat allele of a 30 bp variable number tandem repeat (VNTR) polymorphism located 1.2 kb upstream of the MAO-A coding sequence was reported to be associated with aggression and impulsivity [8]. This finding was subsequently replicated in a subgroup of patients with ADHD and co-morbid conduct disorder [9]. There have however been discrepant results with a report that the longer 4-repeat allele was associated with ADHD [10], a finding that was not replicated in the study from Lawson et al. [9] and a further study from Domschke et al. [11].

Another polymorphism in MAO-A (941G/T), an Fnu4HI G/T polymorphism at position 941 of the protein coding sequence was reported to be associated with high (G-allele) and low (T-allele) MAO-A activity [12]. The high activity G-allele was reported to be associated with ADHD in an Irish sample (χ^2 ^= 5.1, *P *= 0.03. OR = 1.70) and significantly increased transmission of a haplotype consisting of the 3-repeat allele of the promoter VNTR, the 6-repeat allele of a CA microsatellite and the G-allele of the 941G/T to ADHD probands was also reported in [11]. No association with 941G/T SNP was found in the study from Lawson et al. [9].

To provide further clarification of the reported associations in MAO-A we have investigated the two MAO-A markers reported to be associated with ADHD in the previous studies using a sample of 212 ADHD families ascertained in Taiwan.

## Methods

The Taiwanese sample consisted of 212 children with ADHD diagnosed between the ages of 5–15 years. Both parents were available for 114 families, only the mother for 59 families and only the father for 39 families. ADHD cases were ascertained from Child Psychiatric Clinics in the Chang Gung Memorial Hospital in Taipei area, Taiwan. A diagnosis of ADHD was made according to DSM-IV criteria following completion of a standard maternal interview (kiddie-SADS, Kaufman et al., [13]) and completion of parent and teacher Conner's revised rating scales [14]. In all 78% had the combined subtype and 22% the inattentive subtype of ADHD. With regard to co-morbidity, 4% had Tourettes syndrome and 4% oppositional defiant disorder. Autism cases were excluded from the study. No other neurological or behavioural disorders were identified. Eighty-nine percent of the sample was male. The subjects gave their written informed consent, and the study was approved by the Institutional Review Board, Chang Gung Memorial Hospital, Taiwan (Reference number: 94–471).

The MAO-A 30 bp-promoter VNTR was genotyped using the method described in Deckert et al. [15]. As in previous studies [10,9,11] the alleles of the MAO-A 30 bp VNTR were grouped into two classes (short allele: 2, 3; long allele: 3.5, 4 and 5) for the analysis based on the functional roles that the longer alleles are more active than allele 3 [15]. The 941G/T polymorphism was genotyped using Fnu4HI restriction enzyme following the protocol in Domschke et al. [11].

Family genotype data were analysed using the transmission disequilibrium test (TDT) implemented in UNPHASED [16]. Since MAO-A is X-linked gene, genotype data from fathers was only used in the analysis of female ADHD probands. Genotype information from both parents in female probands, and from mother alone in male probands was used to analyse linkage disequilibrium (LD).

## Results

The allele frequency of the two markers was estimated from the probands' mothers. For MAO-A 30 bp VNTR the most common alleles are the 3 and 4 repeats accounting for 98% of all alleles. The 2, 3.5 and 5 repeats are all rare. For MAO-A 941 G/T polymorphism the G-allele frequency is 58% and the T-allele frequency is 42%. TDT analysis (Table 1) showed a significant increase in transmission of the G-allele of 941G/T SNP (χ^2 ^= 4.48, *P *= 0.034, OR = 1.57). In contrast, although the 3-repeat allele of the 30 bp VNTR was over-transmitted to affected offspring, this association was not significant in this sample (χ^2 ^= 2.42, *P *= 0.120, OR = 1.3). We also looked at 4% of the ADHD cases which showed oppositional defiant disorder in our samples. The results revealed that the 4% cases did not drive the nominal association (for 941 G/T SNP: χ^2 ^= 3.0, *P *= 0.08; for 30 bp VNTR: χ^2 ^= 1.0, *P *= 0.31). Due to the differences in the prevalence of ADHD between females and males the sex specific analysis was conducted. In our study 89% of the sample was male. We found that the sex specific analysis for 941G/T SNP did not affect the association results very much (χ^2 ^= 4.1, *P *= 0.04 in male samples; χ^2 ^= 0.33, *P *= 0.56 in female samples). No significant association was found in 30 bp VNTR marker in the sex specific analysis.

**Table 1:** TDT Analysis of MAO-A Markers

	30 bp VNTR						941 G/T	
		
	Allele						Allele	
		
	2R	3R	4R	5R	Short (2R, 3R)	Long (4R, 5R)	G	T
Transmitted	2	52	38	1	54	39	55	35
Non-transmitted	0	40	53	0	39	54	35	55
χ^2^ (*P*-value)					2.42, 1df (0.120)		4.48, 1df (0.034)	

Moderate LD was observed between the two MAO-A markers (D' = 0.62, R^2 ^= 0.60). Analysis of transmission ratios for the common haplotypes of these two alleles revealed a significantly increased transmission of the 3-repeat allele of the 30 bp VNTR and the G-allele of the 941 G/T SNP to ADHD cases (3-G haplotype; χ^2 ^= 4.00, *P *= 0.045, OR = 1.25) and decreased transmission of the common alternative 4-T haplotype (χ^2 ^= 3.92, *P *= 0.048).

An alternative haplotype analysis program, WHAP [17] was used to test the contribution of the individual markers to the haplotype association. WHAP allows the user to drop one or more markers to test whether they contribute significantly to the haplotype association. The results of this analysis showed that the 30 bp VNTR could be dropped and did not make a significant contribution to the haplotype association. In contrast the 941G/T marker made a significant contribution to the haplotype association (dropping the 941G/T,* P *= 0.97).

## Discussion

In the present study, we set out to investigate previously reported findings of association between two genetic variants of the MAO-A gene in a sample of ADHD probands from Taiwan. We found a significant over-transmission of the G-allele of MAO-A 941G/T SNP to ADHD cases. Our finding replicates the results of a recent study conducted by Domschke et al. [11] who found that the more active G-allele of 941G/T SNP was associated with ADHD in an Irish ADHD sample (*P *= 0.03). In addition, Domschke et al. [11] also found a significantly increased transmission of a haplotype containing the 3-repeat allele, the 6-repeat allele of the CA microsatellite and the G-allele of the 941G/T SNP to ADHD cases (*P *= 0.01). In this study we did not genotype CA microsatellite in intron 2 in MAO-A gene, but haplotype analysis of the two markers revealed increased transmission of a haplotype consisting of the 3-repeat allele of the promoter VNTR and the G-allele of the 941G/T SNP to ADHD cases, which was driven by the stronger G allele association.

The association fits with general theories for ADHD. The G-allele of the 941G/T SNP is associated with increased activity of MAO-A and will therefore lead to increased rates of degradation of dopamine and other monoamine neurotransmitters [12]. These data are therefore consistent with an overall hypo-dopaminergic hypothesis for ADHD and previous association findings reported within the ADHD literature. Thus, the 7-repeat allele of DRD4 is thought to be associated with blunted receptor response to dopamine stimulation, and the 10-repeat allele of the dopamine transporter gene is thought to be associated with increased expression of the transporter protein, both hypothesised to result in an overall decrease in availability of or response to dopamine [2].

## Conclusion

In the present study, we investigated the two MAO-A markers reported to be associated with ADHD in the previous studies using a Taiwanese ADHD sample. The findings of this study suggest that the more active MAO-A 941G allele may be a risk factor in the development of ADHD. Due to inconsistencies that remain in the literature and relatively few published data on the two MAO-A polymorphisms investigated in this study, further association studies are needed to confirm or refute these findings.

## Abbreviations

**MAO-A **– monoamine oxidase A, **ADHD **– attention deficit hyperactivity disorder, **TDT **– transmission disequilibrium test, **VNTR **– variable number tandem repeat, **SNP **– single nucleotide polymorphism.

## Competing interests

The author(s) declare that they have no competing interests.

## Authors' contributions

XX selected the markers, carried out the genotyping of the population and performed statistical analysis under the supervision of Professor Philip Asherson. KB and PA helped for revising the paper. CKC, YSH and YYW provided the DNA samples and clinical data. All authors read and approved the final manuscript.

## Pre-publication history

The pre-publication history for this paper can be accessed here:


